# Vitamin D Deficiency Increases the Risk of Adverse Neonatal Outcomes in Gestational Diabetes

**DOI:** 10.1371/journal.pone.0164999

**Published:** 2016-10-20

**Authors:** Letícia Schwerz Weinert, Angela Jacob Reichelt, Leonardo Rauber Schmitt, Roberta Boff, Maria Lucia Rocha Oppermann, Joiza Lins Camargo, Sandra Pinho Silveiro

**Affiliations:** 1 Postgraduate Program in Endocrinology, Universidade Federal do Rio Grande do Sul, Porto Alegre, Brazil; 2 Endocrine Division, Hospital de Clínicas de Porto Alegre, Porto Alegre, Brazil; 3 Medical School, Universidade Federal do Rio Grande do Sul, Porto Alegre, Brazil; 4 Obstetrics Division, Hospital de Clínicas de Porto Alegre, Porto Alegre, Brazil; University of Missouri Columbia, UNITED STATES

## Abstract

**Background:**

Gestational diabetes mellitus (GDM) and vitamin D deficiency have been associated with increased risk of adverse perinatal outcomes but the consequences of both conditions simultaneously present in pregnancy have not yet been evaluated. Our objective was to study the influence of vitamin D deficiency in neonatal outcomes of pregnancies with GDM.

**Methods:**

184 pregnant women with GDM referred to specialized prenatal monitoring were included in this cohort and had blood sampled for 25-hydroxyvitamin D measurement. Vitamin D was measured by chemiluminescence and deficiency was defined as < 20 ng/mL. Participants were followed until puerperium and adverse neonatal outcomes were evaluated.

**Results:**

Newborns of women with vitamin D deficiency had higher incidences of hospitalization in intensive care units (ICU) (32 vs 19%, P = 0.048), of hypoglycemia (any, 17.3 vs 7.1%, P = 0.039requiring ICU, 15.3 vs 3.6%, P = 0.008), and were more frequently small for gestational age (SGA) (17.3 vs 5.9%, P = 0.017). After adjustment, relative risk (RR) for hypoglycemia requiring ICU was 3.63 (95%CI 1.09–12.11) and for SGA was 4.32 (95%CI 1.75–10.66). The incidence of prematurity, jaundice and shoulder dystocia was no statistically different between groups.

**Conclusions:**

In this cohort of pregnant women with GDM, vitamin D deficiency was associated with a major increase in the incidence of adverse neonatal outcomes such as SGA newborns and neonatal hypoglycemia.

## Introduction

Several physiologic adaptations of the calcium and vitamin D metabolism are necessary during pregnancy in order to provide sufficient nutrients to the fetus and the neonate[1]. Fetuses depend entirely on the mother as a source of vitamin D and plasma levels of 25-hydroxyvitamin D (25(OH)D) in the newborn average 60 to 70% of the maternal level[2]. However, pregnant women have an unacceptably high prevalence of vitamin D deficiency and insufficiency[2–4], and this has been reported to increase the risk of adverse maternal and neonatal outcomes[5].

Studies have demonstrated that lower maternal levels of 25(OH)D result in reduction of birth weight and that vitamin D deficiency is associated with a higher risk of neonates small for gestational age (SGA)[6–11]. Reduced intrauterine long bone growth may also occur[12,13], and poor skeletal mineralization and lower bone mineral content have been detected in the children[14] of vitamin D deficient mothers. Other possible major perinatal and childhood outcomes related to vitamin D deficiency status in pregnancy are respiratory tract infections, type 1 diabetes and central nervous system disorders[5,15].

Gestational diabetes (GDM) is another common condition associated with adverse perinatal outcomes, such as macrosomia, neonatal hypoglycemia and shoulder dystocia[16]. Moreover, long-term evaluation reveals an increased rate of adverse outcomes such as type 2 diabetes and pre-diabetes in the adult offspring of women with GDM [17].

Therefore, our objective was to investigate the neonatal complications of vitamin D deficiency in the presence of GDM.

## Materials and Methods

### Study population and research design

The study protocol and the informed consent form were approved by the Ethics Committee of Hospital de Clínicas de Porto Alegre. Participants received information about the study in order to enable the voluntary decision to participate or not as research subjects; all of the pregnant adult women included in the study provided written informed consent; we did not included minors.

The multidisciplinary team specialized in the care and research of diabetes during pregnancy at Hospital de Clínicas de Porto Alegre assists pregnant women with hyperglycemia referred to high risk prenatal monitoring. Between November 2009 and May 2012, we invited all women with a diagnosis of GDM to participate in the study. They answered a questionnaire requesting information on socio-demographic variables, medical history and pregnancy. Physical examination was performed at all prenatal visits. Blood was sampled after 8 hours fasting in the third trimester of pregnancy since the diagnosis of GDM is usually made after the 24^th^ week of gestation. This cohort was prospectively followed until the puerperium period and the newborns of participants were evaluated.

The diagnosis of GDM at our institution follows the recommendations of the 2^nd^ Meeting of The Diabetes and Pregnancy Task Force[18]: a 75g oral glucose tolerance test (75g OGTT) was performed if the screening was positive (fasting plasma glucose ≥ 85 mg/dL [4.7 mmol/L]) and gestational diabetes was defined by the cut off point of fasting plasma glucose ≥ 110 mg/dL (6.1 mml/L) or 2h plasma glucose ≥ 140 mg/dL (7.8 mmol/L). After the publication of the diagnostic proposal of the International Association of Diabetes and Pregnancy Study Groups (IADPSG) and its adoption by the American Diabetes Association (ADA)[19], we also adopted this criteria and women who had fasting plasma glucose ≥ 92 mg/dL (5.1 mmol/L) or 1h plasma glucose ≥ 180 mg/dL (10 mmol/L) or 2h plasma glucose ≥ 153 mg/dL (8.5 mmol/L) after the 75g load was included in the study.

### Covariates

Several clinical variables were evaluated at the maternal interview: maternal age, self-reported skin color (white or dark skin tone), socioeconomic status (Brazilian classification[20]; Status A to E), years of study, parity, self-reported pre-pregnancy weight, active smoking, chronic disease, use of medicines and supplements, parity, previous history of a child with macrosomia, GDM, and gestational hypertension or preeclampsia/eclampsia. Height was measured at first prenatal visit and body mass index (BMI) was calculated as pre-pregnancy weight/height^2^ (kg/m^2^). Routine prenatal laboratory tests and HbA1c were evaluated. Data about delivery route, medical emergencies, neonatal intensive care unit (ICU) hospitalization, neonatal weight immediately after birth, sex and health status were extracted from medical records.

### Outcomes

We evaluated the following neonatal outcomes: birth weight, incidence of newborn SGA or large for gestational age (LGA) (< percentile 10 and > percentile 90 for sex and gestational age, respectively[21]), neonatal hypoglycemia (glucose < 45 mg/dL) with or without ICU hospitalization, prematurity (gestational age <37 weeks), ICU hospitalization for any cause, jaundice, shoulder dystocia, and death (fetal or neonatal).

Gestational age was based on an ultrasound early in pregnancy. Adequacy of birth weight for gestational age was based on the traditional curve of Alexander et al[21], but we also analyzed the results for the recently published Brazilian charts[22].

### Laboratory

Glucose was measured by the colorimetric method (Advia 1800, Siemens Healthcare, Erlangen, Germany) and HbA1c by high performance liquid chromatography (Variant II, BioRad Laboratories, Hercules, CA). Serum 25(OH)D was measured by chemiluminescent immunoassay (DiaSorin LIAISON^®^, MN, USA). This assay measures between 4.0 and 150 ng/mL, and inter-assay precision had a coefficient of variation of < 4% and < 6% for 15 and 50 ng/mL values, respectively. Vitamin D deficiency was defined as 25(OH)D below 20 ng/mL, in agreement with current recommendations of the Institute of Medicine[23] and the Endocrine Society[24].

Maternal blood was sampled just at one time during the third trimester of pregnancy. Serum for 25(OH)D measurement was prepared by centrifugation and was stored at -80°C until analysis. After delivery, 25(OH)D was measured and women were classified according to the presence of vitamin D deficiency (risk group) or not (control). Blood was sampled at all seasons. Season was categorized by winter (June 21 –September 22), spring (September 23 –December 20), summer (December 21 –March 20), and autumn (March 21 –June 20). Porto Alegre city is located at latitude 30°S.

### Statistical analysis

Descriptive statistics were used to present the data about clinical and demographic variables of mother and neonate in each vitamin D category (below or above 20 ng/mL). Number, percentage, mean ± standard deviation, median and interquartile range were used.

Comparison between risk and control groups was performed. Categorical variables were analyzed using the chi-square test, and continuous variables were evaluated with the t-test when parametric and with the Mann-Whitney test when non-parametric. A general mixed model (Poisson regression with robust standard errors) was used to study the association of SGA, neonatal hypoglycemia, and ICU hospitalization with serum vitamin D status. Adjustment models took into consideration potential biological confounders such as maternal glucose at OGTT, HbA1c, maternal BMI, skin pigmentation, birth weight, gestational age, smoking, nulliparity, hypertensive disorders of pregnancy (gestational hypertension and preeclampsia), medication requirement for diabetes treatment, maternal age, low socioeconomic status, years of study and season. Confounding factors were considered not to influence the main result and were removed from the model if their inclusion did not change relative risk (RR) by at least 5%. Correlations were evaluated with Pearson´s or Spearman´s test for parametric and non-parametric variables, respectively. Analyses were also performed between ethnic subgroups.

The required number of newborns to be included in the cohort was 158, based on a 7% prevalence of SGA[25] and on a previous published odds ratio of 3.17[7]. Statistical analysis was performed in SPSS software version 20.0 and statistical significance was considered when P<0.05.

The reporting of this study is in accordance with the Strobe Statement[26].

## Results

**Fig 1:**
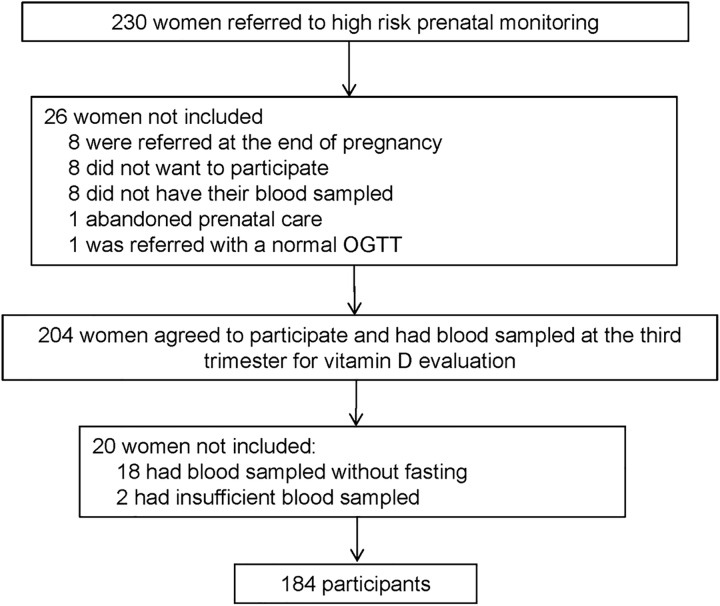
Flowchart of the study.

Two hundred and thirty women with GDM were referred to high risk prenatal monitoring and 184 of them were included in this cohort (Fig 1). Their blood was sampled for storage and they were followed until postpartum and newborn evaluation. Maternal clinical and laboratory characteristics are shown in Table 1. Ninety-eight women (53.3%) had vitamin D deficiency. No woman was regularly taking vitamin D supplement.

**Table 1:** Clinical and laboratory characteristics of the 184 women with gestational diabetes.

	25(OH)D ≥ 20 ng/mL	25(OH)D < 20 ng/mL	P
	N = 86	N = 98	
25(OH)D (ng/mL)	27.4 ± 4.8	12.9 ±4.4	<0.001
Season* (n,%)			<0.001
Summer	47 (54.7)	11 (11.2)	
Autumn	25 (29.1)	32 (32.7)	
Winter	3 (3.5)	22 (22.4)	
Spring	11(12.8)	33 (33.7)	
Age (years)	32.0 ± 6.3	32.3 ± 5.8	0.744
White (n,%)	69 (80.2)	67 (68.4)	0.048
Eight years of schooling (n,%)	58 (67.4)	64 (66.0)	0.834
Socioeconomic class A or B (n,%)	17 (28.3)	28 (34.6)	0.432
Parity (n)	2 (2–4)	3 (2–4)	0.505
Previous HDP (n,%)	20 (23.3)	20 (20.4)	0.640
Hypertension	20 (23.3)	14 (14.3)	0.130
Preeclampsia or eclampsia	15 (17.5)	16 (16.4)	0.592
Chronic Hypertension (n,%)	5 (5.8)	12 (12.2)	0.130
Smoking (n,%)	9 (10.5)	12 (12.2)	0.705
BMI (kg/m^2^)	29.1 ± 6.6	30.2 ± 6.9	0.290
75g OGTT (mg/dL; mmol/L)			
Fasting glucose	97.8 ± 26.1	102.8 ± 27.8	0.215
	(5.4 ± 1.4)	(5.7 ±1.5)	
1h glucose^†^	177.0 ± 32.5	178.2 ± 27.7	0.924
	(9.8 ± 1.8)	(9.9 ± 1.5)	
2h glucose	165.4 ± 25.5	160.8 ± 27.5	0.269
	(9.2 ± 1.4)	(8.9 ± 1.5)	
HbA1c (%; mmol/L)^‡^	5.6 (5.1–6.0)	5.6 (5.3–6.1)	0.756
	38 (32–42)	38 (34–43)	

Data expressed as number (%), mean±sd, median (interquartile range); Chi-square test was used for categorical variables, t-test for continuous variables when parametric and Mann-Whitney test when non-parametric; 25(OH)D: 25-hydroxyvitamin D at the third trimester of pregnancy; HDP: Hypertensive disorders of pregnancy; BMI: Body mass index; OGTT: Oral glucose tolerance test

*Season of blood sampling

^†^ 1h glucose is available for only 24 women

^‡^HbA1c at beginning of the third trimester

Table 2 shows neonatal adverse outcomes according to vitamin D status. There was just one fetal death at 33 weeks of pregnancy, in a woman who developed gestational hypertension and preeclampsia; her serum 25(OH)D was 16.2 ng/mL. Three neonatal infections were diagnosed as pneumonia, meningitis and neonatal septicemia; all infections occurred in the vitamin D deficiency group. Neonatal ICU hospitalization was more frequent in the vitamin deficiency group and it was necessary for various neonatal disorders such as jaundice with phototherapy, severe hypoglycemia, grunting, infection, tachypnea of the newborn, prematurity and other less frequent reasons. Table 3 shows the neonatal outcomes after adjustment for significant confounders. Only SGA newborns and neonatal hypoglycemia requiring ICU hospitalization remained associated with lower vitamin D levels.

**Table 2:** Neonatal outcomes according to maternal vitamin D status.

	25(OH)D ≥ 20 ng/mL	25(OH)D < 20 ng/mL	*P*
	N = 86	N = 98	
Gestational age (weeks)	38.4 (38.0–38.9)	38.6 (37.6–39)	0.552
Prematurity (n,%)	10 (11.8)	19 (19.4)	0.159
Birth Weight (g)	3260 ± 535	3179 ± 586	0.334
SGA (n,%)	5 (5.9)	17 (17.3)	0.017
LGA (n,%)	8 (9.4)	8 (8.2)	0.766
Neonatal hypoglycemia (n,%)			
Any hypoglycemia	6 (7.1)	17 (17.3)	0.039
Requiring ICU	3 (3.6)	15 (15.3)	0.008
Jaundice (n,%)			
Any jaundice	15 (17.9)	24 (24.7)	0.261
With phototherapy	9 (10.7)	17 (17.5)	0.193
Shoulder dystocia (n,%)	3 (3.6)	7 (7.1)	0.292
Neonatal infection (n,%)	0	3 (3.1)	-
ICU hospitalization* (n,%)	16 (19)	31 (32)	0.048
Death (n,%)	0	1 (1)	-

Data expressed as number (%), mean±sd, median (interquartile range); Chi-square test was used for categorical variables, t-test for continuous variables when parametric and Mann-Whitney test when non-parametric; 25(OH)D: 25-hydroxyvitamin D; SGA: Small for gestational age; LGA: Large for gestational age; ICU: Intensive care unit

*ICU hospitalization for any cause

**Table 3:** Results of Poisson regression analysis for neonatal outcomes.

	RR, crude	95% CI	RR, adjusted	95% CI
SGA* (n,%)	2.95	1.14–7.66	4.32	1.75–10.66
Neonatal hypoglycemia^†^ (n,%)				
Any hypoglycemia	2.43	1.00–5.88	2.40	0.90–6.42
Requiring ICU	4.29	1.29–14.30	3.63	1.09–12.11
ICU hospitalization^‡^ (n,%)	1.68	0.99–2.85	1.31	0.70–2.42

25(OH)D: 25-hydroxyvitamin D; SGA: small for gestational age; ICU: intensive care unit

*Adjusted for maternal glucose at OGTT, maternal body mass index, smoking and season

^†^Adjusted for maternal glucose at OGTT, birth weight, gestational age and season

^‡^Adjusted for maternal glucose at OGTT, maternal body mass index, birth weight, gestational age, and season

**Fig 2:**
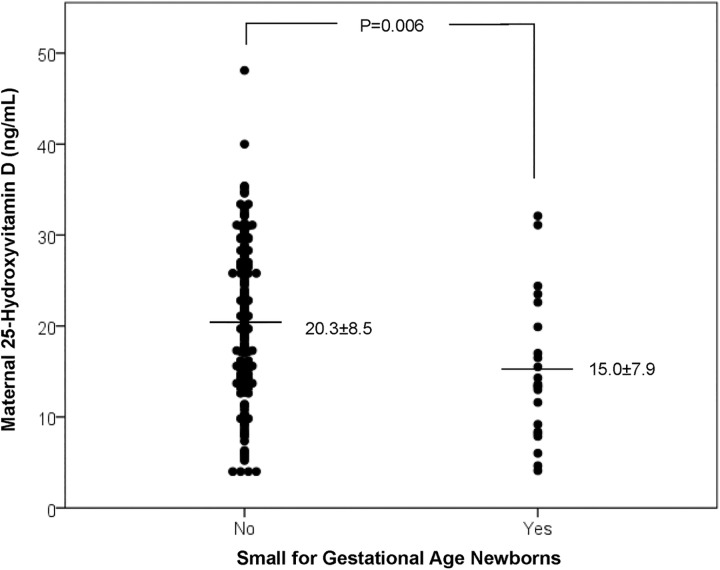
Maternal vitamin D in newborns small for gestational age or not.

The incidence of SGA newborns was higher in the group of vitamin D deficient women (Table 2). The analysis of SGA newborns with the charts of Alexander et al presented an RR for vitamin D deficiency of 4.32 (95%CI 1.75–10.66) after adjustments (Table 3), while the analysis with the Brazilian chart showed a similar significant RR of 4.55 (95%CI 1.30–16.04). When analysis was performed in order to compare the groups of newborns who were SGA or not, we detected a lower 25(OH)D level in the SGA group (Fig 2).

Prematurity rates were not statistically different between the group with or without vitamin D deficiency (Table 2) and serum 25(OH)D did not correlate with gestational age at delivery (P = 0.695). On the other hand, the analysis of newborns with or without premature delivery showed a trend for lower serum vitamin D in preterm births (20.2±8.5 vs 16.9±8.4 ng/mL, P = 0.059).

Analysis of data considering maternal skin pigmentation differences, showed an incidence of SGA of 9.6% in white and 18.8% in dark-skinned toned women (P = 0.095). In white women, the vitamin D deficient group had a higher incidence of SGA (16.4% vs 2.9%, P = 0.008). The RR for SGA in white women with 25(OH)D < 20 ng/mL was 6.04 (CI95% 1.90–19.23) after adjustment for maternal glucose at OGTT, maternal body mass index, smoking and season. However, in dark-skinned women, there was no difference in the rates of SGA according to vitamin D status (19.4% in the deficient group vs 17.6% in the group with 25(OH)D ≥ 20 ng/mL, P = 0.885).

## Discussion

Maternal vitamin D deficiency was associated with increased rates of neonatal hypoglycemia requiring ICU hospitalization and of SGA newborns in our cohort of pregnant women with GDM. Many previous studies have reported an increased risk of SGA newborns in general women with deficiency of vitamin D[6–9,11]. This finding is consistent in different populations and variable trimester of 25(OH)D measurement. A large multi-ethnic cohort in The Netherlands evaluated 3730 relatively healthy women with measurement of vitamin D in early pregnancy and found that deficient vitamin D levels resulted in an odds ratio (OR) of 2.4 (CI95% 1.9–3.2) for SGA[9]. In a U.S. cohort, maternal vitamin D deficiency in the first trimester was also associated with higher SGA incidence, and birth weight increased with rising levels of 25(OH)D[8]. In another cohort, with second trimester plasma 25(OH)D measurement, lower levels of vitamin D were associated with higher odds of SGA[7]. In another study with ethnically diverse patients, women with vitamin D insufficiency and deficiency evaluated around the 15^th^ week of pregnancy had neonates with lower birth weight than patient with vitamin D levels above 30 ng/mL (139.74 g and 175.52 g, respectively)[11]. A recent meta-analysis on this issue reinforced the association[10]. In agreement with these previous data in general pregnancies, we found a major increase in the incidence of SGA neonates in pregnancies with GDM. Therefore, as far as we know, we are the first to show this association in a population of diabetic women whose offspring are at higher risk of being LGA. Moreover, few studies have measured vitamin D at the third trimester, when the fetus grows faster.

Since vitamin D deficiency is more prevalent in dark-skinned women, it could be one of the possible mechanisms to explain ethnic differences in neonatal outcomes. However, available data are not conclusive. In our cohort, vitamin D influenced the SGA incidence in white women, but it did not change the risk in non-white women. Previous studies also did not find any relationship between serum 25(OH)D and SGA risk in black women[6] or described a limited role of vitamin D in the explanation of multi-ethnic differences[9]. In contrast, Burris et al demonstrated a higher incidence of SGA in black women and they raised the possibility that vitamin D might contribute to racial disparities [7].

The biological mechanism of vitamin D influence on fetal growth is still not fully understood. One of the plausible explanations is that maternal vitamin D deficiency may result in abnormal maternal calcium metabolism, and lower calcium levels in cord blood are associated with lower birth length[27]. Another speculated mechanism is that vitamin D might regulate glucose metabolism[28], which affects fetal mass. Moreover, human placenta may have a role in the influence of vitamin D on fetal growth since vitamin D receptors and enzymes to convert 25(OH)D in 1,25 dihydroxyvitamin D have been identified in the placenta tissue, and calcitriol may regulate human chorionic gonadotropin and sex steroid secretion [29,30].

It is also important to highlight that intrauterine growth restriction is a relevant neonatal outcome, since it results in increased fetal and perinatal morbidity and mortality [31]. Moreover, fetal origins of adult disease theory assumes that SGA infants are at increased risk for adult coronary heart disease and related disorders. A large cohort reported an increased risk of ischemic heart disease mediated by poor fetal growth[32]. Therefore, fetal growth restriction is associated with an increased rate of complications early or late in life, and further longitudinal studies are needed to completely understand the long-term consequences of SGA neonates.

Transient asymptomatic hypoglycemia may be part of the transition to extrauterine environment, but severe or persistent neonatal hypoglycemia could result in ICU hospitalization, higher cost and neurologic damage. This is the first study to show the association of vitamin D deficiency and increased rates of neonatal hypoglycemia. The association is significant for any hypoglycemia but, more importantly, we also show an association of vitamin D deficiency with severe hypoglycemia requiring ICU hospitalization. This is a relevant outcome in an at risk population because they are newborns of women with GDM. Further studies are necessary to reinforce this association.

The incidence of prematurity was higher in the pregnancies with vitamin D deficiency and serum vitamin D was lower in the group with preterm newborns, although there was no statistically significant difference in our study. Some other studies did not find a lower gestation age at birth in pregnancies complicated by vitamin D deficiency[7,33] while other data reported an association[9,13]. A recent meta-analysis found that maternal vitamin D deficiency increases the risk of preterm birth[34].

Based on observational study findings, vitamin D supplementation has been recommended during pregnancy. Two trials from the 1980s reported a lower incidence of SGA neonates[35] and a better birthweight[36] in mothers who had vitamin D supplementation. Since that, many trials of vitamin D supplementation during pregnancy have been published and a meta-analysis of randomized controlled trials[37] concluded that birth weight and birth length were greater for neonates in the vitamin D supplementation group, but authors did not find a difference for low birth weight, SGA and prematurity incidences. A recent Cochrane review[38] reported that supplementation with vitamin D alone (without calcium), with respect to infant outcomes, decrease the risk of preterm birth rates and birthweight below 2500 g.

This cohort study adds novel data about vitamin D deficiency in a group of women with GDM. This population has a high incidence of vitamin D deficiency and it is associated with increased rates of adverse neonatal outcomes. The strengths of our study are the prospective design and novel data in a previously not studied group of DMG women. Moreover, we used both a standard international chart for birthweight analysis and the recently built national chart. Relative risk for SGA was very similar between the charts, which reinforced our findings. However, this study also has some limitations. First, we have not assessed calcium intake or serum level. It is relevant since calcium levels in cord blood have been reported to be associated with birth length[27]. Second, serum vitamin D binding protein was not measured and therefore we were not able to estimate free 25(OH)D, but we understand that rates of vitamin D deficiency would be, indeed, at most underestimated and would not invalidate the results. Third, we used both the Brazilian and the ADA recommendations for GDM diagnosis, since there was no current consensus.

## Conclusion

In conclusion, vitamin D deficiency was associated with an increased incidence of adverse neonatal outcomes, such as neonatal hypoglycemia requiring ICU and SGA newborns, in our cohort of high-risk pregnant women with GDM. We consider it is imperative to confirm our findings in other independent large cohorts. Vitamin D is a modifiable risk factor in pregnancy with important public health implications and we believe that national authorities should implement routine vitamin D measurement during pregnancy.
